# Photosynthetic response dynamics in the invasive species *Tithonia diversifolia* and two co-occurring native shrub species under fluctuating light conditions

**DOI:** 10.1016/j.pld.2023.04.001

**Published:** 2023-04-25

**Authors:** Ju Li, Shu-Bin Zhang, Yang-Ping Li

**Affiliations:** a CAS Key Laboratory of Tropical Forest Ecology, Xishuangbanna Tropical Botanical Garden, Chinese Academy of Sciences, Menglun, Mengla 666303, Yunnan, China; b University of Chinese Academy of Sciences, Beijing 100049, China

**Keywords:** Invasive plant, Photosynthetic induction, Photosynthetic relaxation, Carbon gain, Stomatal traits, *Tithonia diversifolia*

## Abstract

To determine the invasiveness of invasive plants, many studies have compared photosynthetic traits or strategies between invasive and native species. However, few studies have compared the photosynthetic dynamics between invasive and native species during light fluctuations. We compared photosynthetic induction, relaxation dynamics and leaf traits between the invasive species, *Tithonia diversifolia* and two native species, *Clerodendrum bungei* and *Blumea balsamifera*, in full-sun and shady habitats. The photosynthetic dynamics and leaf traits differed among species. *T. diversifolia* showed a slower induction speed and stomatal opening response but had higher average intrinsic water-use efficiency than the two native species in full-sun habitats. Thus, the slow induction response may be attributed to the longer stomatal length in *T. diversifolia*. Habitat had a significant effect on photosynthetic dynamics in *T. diversifolia* and *B. balsamifera* but not in *C. bungei*. In shady habitat, *T. diversifolia* had a faster photosynthetic induction response than in full-sun habitat, leading to a higher average stomatal conductance during photosynthetic induction in *T. diversifolia* than in the two native species. In contrast, *B. balsamifera* had a larger stomatal length and slower photosynthetic induction and relaxation response in shady habitat than in full-sun habitat, resulting in higher carbon gain during photosynthetic relaxation. Nevertheless, in both habitats, *T. diversifolia* had an overall higher carbon gain during light fluctuations than the two native species. Our results indicated that *T. diversifolia* can adopt more effective response strategies under fluctuating light environments to maximize carbon gain, which may contribute to its successful invasion.

## Highlights

• Habitat affected photosynthetic response dynamic but responses to the driver are species specific.
• Photosynthetic induction response strategy of *Tithonia diversifolia* is more efficient relative to the two native species.
• Difference in photosynthetic response dynamic were associated with stomatal morphological traits.

## 1 Introduction

Biological invasion is a key threat to global biodiversity and ecosystem function (Diagne et al., 2021). Invasive plants may have different functional traits or strategies that allow them to succeed in the introduced ranges. Among these, photosynthesis is generally regarded as an important process for supporting plant growth and received more attention. Over the last decade, many studies have compared steady-state photosynthetic processes between invasive and native species. However, the light intensity in the field frequently changes from shade to sun and sun to shade in seconds (Pearcy, 1994; Zhu et al., 2004; Slattery et al., 2018). Fluctuating light may lead to dynamic photosynthesis and influence total carbon gain in the field (Pearcy, 1990). Modulating photosynthesis under fluctuating light conditions may more accurately reflect the photosynthetic process in the field. However, few studies have focused on comparing photosynthetic dynamics between invasive and native species under fluctuating light conditions.

The process by which leaves begin to increase the assimilation of CO_2_ as light transitions from low (shade) to high (sun) is defined as photosynthetic induction, which features a lag due to time required for the regeneration of ribulose 1,5-bisphosphate (RuBP), the synthesis of carbon metabolite intermediates, activation of ribulose 1,5-bisphosphate carboxylase/oxygenase (Rubisco), and stomatal opening when photosynthesis shifts to a steady state (Pearcy, 1994; Mott and Woodrow, 2000). Because of the delay effect, the leaf photosynthetic rate (*A*) is lower throughout induction than at steady state, which results in a considerable loss of daily carbon gain (Taylor and Long, 2017; Tanaka et al., 2019). Electron transport and activation have been shown to limit CO_2_ assimilation (*A*) during short flecks (Soleh et al., 2017; Taylor and Long, 2017) because of the rapid activation of RuBP regeneration and Rubisco (Mott and Woodrow, 2000; Kaiser et al., 2016; Deans et al., 2019). In addition, diffusion triggered by stomatal conductance (*gs*) and mesophyll conductance (*gm*) mainly constrains *A* during longer light periods (Pearcy, 1990). Both *gs* and *gm* influence dynamic photosynthesis under light fluctuation and, in turn, affect CO_2_ provision to the RuBP carboxylation site (Sakoda et al., 2022).

Leaf stomata predominantly control CO_2_ uptake for photosynthesis and transpiration and further determine plant productivity and water-use efficiency. The balance between photosynthesis and transpiration relies on internal signals and environmental sensitivity of stomata, and the synchrony of stomatal movement relative to the CO_2_ requirement of the mesophyll (Lawson and Blatt, 2014). The response of *gs* to light fluctuation is slower commonly than the biochemical process, which is a significant limitation of *A* during the induction phase (Kaiser et al., 2017; Sakoda et al., 2022). The response of *gs* is primarily explained by the stomatal movement via the regulation of signal transduction and metabolic processes (Lawson and Blatt, 2014). *gs* is also affected by the morphological features of the stoma, such as the size and shape (dumbbell and elliptical shaped) of a single stoma, stomatal density, and stomatal distribution (Sakoda et al., 2022). Several studies have shown that smaller stomata have faster response rates than larger ones (Drake et al., 2013; Lawson and Blatt, 2014). However, it is remains controversial. Other studies have not observed a relationship between stomatal behavior and morphology (Elliott-Kingston et al., 2016). Zhang et al. (2019) found that smaller stomata have a slower initial induction speed and lower stomatal conductance during the initial phase of light induction relative to larger stomata.

*Tithonia diversifolia* A. Gray, a perennial invasive shrub, is native to Central America (Laduke, 1982). It was introduced as an ornamental and green manure plant in many countries, which offered opportunities for its dispersal throughout most of the tropical and subtropical areas worldwide (Morales, 2000). This species can reproduce both sexually, with a large production of seeds, and asexually. Once its population is established, it quickly forms dense monospecific stands by inhibiting the germination and growth of neighboring species (Oyerinde et al., 2009; Otusanya and Ilori, 2012; Kato-Noguchi, 2020), leading to a decrease in biodiversity (Obiakara and Fourcade, 2018; Dai et al., 2021). It can invade a variety of habitats. It can grow in open and sunny areas, such as roadsides, wastelands, riverbanks and disturbed sites, and in shady areas, including forest edges and disturbed secondary forests. This suggests that they are likely to respond rapidly and efficiently to heterogenous environments during physiological processes. However, the photosynthetic strategy of *T. diversifolia* in different habitats under natural fluctuating light conditions remains unclear.

Here, we measured gas exchange under fluctuating light conditions in *Tithonia* *diversifolia* and two native species, *Clerodendrum bungei* and *Blumea balsamifera*, grown in full-sun and shady sites. We selected these two native plants because of their overlapping habitats. *C. bungei* is an accompanying species of *T. diversifolia*; *B. balsamifera*, similar to *T. diversifolia*, belongs to the Compositae family. We also measured the stomatal traits of all species. We hypothesized that (1) *T. diversifolia* has a faster and more efficient response to photosynthetic induction than two native species, (2) habitat type under different light intensities affects photosynthetic dynamics, and (3) changes in photosynthetic induction and relaxation speed are associated with leaf traits.

## 2 Materials and methods

### 2.1 Study site and species

This study was conducted at Xishuangbanna Tropical Botanical Garden (XTBG), Chinese Academy of Sciences, Yunnan Province, China. The mean day/night temperature in March at XTBG is 22.8 °C/15.2 °C, with a day/night relative humidity of 64%/100% (Liu et al., 2004). Our study included an invasive shrub, *Tithonia diversifolia*, and two native shrubs, *Clerodendrum* *bungei* and *Blumea* *balsamifera*. Prior to this study, *T. diversifolia* and *C*. *bungei* grew naturally in XTBG, and *B*. *balsamifera* was planted for more than 20 years in XTBG. These three species can grow under full-sun light and shady conditions in the field. We identified six locations with similar soil but different light conditions under which the three species grew (see Table 1). For each individual, similar mature leaves (4th–5th from the shoot tip) in the new shoots of the current year were selected.

**Table 1:** Background information of sampling sites.

Habitat	Species	Height (cm)	Aboveground biomass (g)	Soil pH	Soil AN (mg kg^−1^)	Soil AP (mg kg^−1^)	Soil AK (mg kg^−1^)
Full sun	*T. diversifolia*	427 ± 20^a^	1168.20 ± 92.50^a^	7.77 ± 0.13^a^	12.13 ± 1.83^a^	29.53 ± 6.81^a^	376.83 ± 31.89^a^
	*C. bungei*	414 ± 16^ab^	606.07 ± 53.13^b^	7.55 ± 0.19^ab^	11.11 ± 1.72^a^	33.02 ± 7.78^a^	296.67 ± 45.19^a^
	*B. balsamifera*	155 ± 11^d^	292.65 ± 39.98^c^	7.11 ± 0.08^b^	8.95 ± 0.69^a^	13.51 ± 3.03^b^	212.67 ± 50.44^a^
Shade	*T. diversifolia*	407 ± 7.0^b^	384.09 ± 35.43^c^	7.87 ± 0.07^a^	7.79 ± 1.32^a^	8.17 ± 1.58^b^	281.50 ± 45.86^a^
	*C. bungei*	374 ± 10^c^	536.61 ± 56.75^b^	7.20 ± 0.32^b^	11.03 ± 3.33^a^	20.90 ± 4.22^ab^	365.83 ± 121.93^a^
	*B. balsamifera*	59 ± 3^e^	97.55 ± 13.33^d^	7.10 ± 0.08^b^	11.03 ± 0.54^a^	11.51 ± 3.13^b^	210.07 ± 23.44^a^

Note: All data are mean ± SE of six individuals.

Soil AN, Soil AP, Soil AK mean soil available nitrogen, phosphorus and kalium, respectively.

Different lowercase letters indicate statistically significant difference (*P* < 0.05).

### 2.2 Gas exchange measurements

Gas exchange parameters of six individuals per species were measured under full sunlight (≥ 1200 μmol photons m^−2^ s^−1^) and shade (≤ 300 μmol photons m^−2^ s^−1^) environments using the Li-6800 portable photosynthesis system (LI-COR Inc., USA) with 27 °C air temperature, 500 μmol s^−1^ flow rate, 1.2–1.8 kPa water vapor pressure deficit (VPD), and 65% relative humidity. The mature fully expanded leaves were selected at random for measuring gas exchange parameters, and the measurement was performed between 10:00–14:00 (because of thick fog before 9:30 a.m.) to avoid confounding photosynthesis with any marked circadian effects. The definitions and abbreviations of all the measured traits are specified in Table 2.

**Table 2:** A summary of all traits measured and mentioned in the text. Units and light conditions are included.

	Traits	Description	Unit
**Photosynthetic traits**	**LCP**	Light compensation point	μmol m^−2^ s^−1^
	**LSP**	Light saturation point	μmol m^−2^ s^−1^
	***IT*_50i_**	Time to 50% induction	minute
	***Tgs*_50i_**	Time to 50% stomatal conductance during photosynthetic induction	minute
	***Tgs*_50r_**	Time to 50% stomatal conductance during photosynthetic relaxation	minute
	***gs***_**mi**_	Average stomatal conductance during photosynthetic induction	mol m^−2^ s^−1^
	***gs***_**mr**_	Average stomatal conductance during photosynthetic relaxation	mol m^−2^ s^−1^
	**iWUE**_**mi**_	Average intrinsic water-use efficiency during photosynthetic induction (iWUE = A/*gs*)	μmol CO_2_ mol H_2_O^−1^
	**iWUE**_**mr**_	Average intrinsic water-use efficiency during photosynthetic relaxation (iWUE = A/*gs*)	μmol CO_2_ mol H_2_O^−1^
	***C***_**indu**_	The integrated amount of CO_2_ assimilation during photosynthetic induction	μmol m^−2^
	***C***_**relax**_	The integrated amount of CO_2_ assimilation during photosynthetic relaxation	μmol m^−2^
	***C***_**total**_	The integrated amount of CO_2_ assimilation during photosynthetic induction and relaxation	μmol m^−2^
**Stomatal traits**	**SD**	Stomatal density	No. mm^−2^
	**SL**	Stomatal length	μm
	**SW**	Stomatal width	μm
	**LN**	Leaf nitrogen content	g kg^−1^

To determine the range of adaptation to light intensity, we measured the light response curves of the three species. Photosynthetic parameters were measured at saturating CO_2_ concentration (400 μmol mol^−1^) and light intensities of 0, 15, 75, 150, 200, 300, 500, 800, 1000, 1500, and 2000 μmol m^−2^ s^−1^. The light saturation point (LSP) and light compensation point (LCP) were fitted using “Photosynthesis” v.1.0.

For induction, leaves were allowed to reach a steady state under low light (50 μmol m^−2^ s^−1^ PPFD) for 5 min, followed by 30 min of high light (1700 μmol m^−2^ s^−1^ PPFD). For relaxation, the light intensity on leaves was decreased back to low light (50 μmol m^−2^ s^−1^ PPFD) for 15 min after 30 min of high light. Gas exchange parameters were logged every minute.

Average stomatal conductance (*gs*_mi_) and average intrinsic water-use efficiency (iWUE_mi_) over 30 min of photosynthetic induction and average stomatal conductance (*gs*_mr_) and average intrinsic water-use efficiency (iWUE_mr_) over 15 min of photosynthetic relaxation were calculated.

The speed of photosynthetic induction was calculated by measuring the time in minutes to 50% relative to steady-state CO_2_ uptake (*IT*_50i_). The speed of stomatal opening was assessed as the time to 50% steady-state stomatal conductance during induction (*Tgs*_50i_). The initial *gs* at pre-illumination can constrain the photosynthetic induction response by affecting the activation state of the biochemical response (Kaiser et al., 2016). To eliminate the influence of initial status on induction speed, we regarded the last *A* and *gs* at low light as the initial *A*_0_ and *gs*_0_, and we obtained values of each corresponding Δ*A* and Δ*gs* by subtracting the initial *A*_0_ and *gs*_0_ from the observed value during induction. Finally, we calculated the induction rate of *A* and the opening speed of the stoma with series Δ*A* and Δ*gs*. The speed of stomatal closure was also evaluated based on the time to 50% of the new steady-state stomatal conductance after returning to low light (*Tgs*_50r_).

The integrated amount of CO_2_ uptake (*C*_indu_) during 30 min of light induction, and the integrated amount of CO_2_ uptake (*C*_relax_) during 15 min of light relaxation were calculated using Eq. (1):

(1) $$\left[C=A_{t}\times d_{t}\right]$$

where *A*_t_ is the transient photosynthetic rate (Xiong et al., 2022).

The integrated amount of CO_2_ uptake (*C*_total_) during light induction and relaxation is the sum of *C*_indu_ and *C*_relax_.

### 2.3 Leaf traits measurement

Mature leaves from the same position were sampled. Leaf nitrogen (N, g kg^−1^) concentration was measured using a C–N elemental analyzer (Vario Max CN, Elementar Analysensysteme GmbH, Hanau, Germany). The leaves (2 × 2 cm) were excised and soaked in a solution (ethanol : acetic acid = 1:1, v/v) in a 70 °C thermostatic water bath until the leaves were clear, and the abaxial side of the leaf was torn and stained with safranin. Temporary slices for stomatal observations were made. Images were obtained using a light microscope (Leica Microsystems Ltd, Leica DM2500) connected to a digital camera. The stomatal length and width were measured as the major and minor axes of the ellipse, respectively. Stomatal density was defined as the number of stomata per unit of leaf area.

### 2.4 Statistical analyses

We used a two-way ANOVA to determine the effects of species, habitat type, and their interaction on all traits. Further multiple comparisons among species and habitat types were performed using the new least significant range method (Duncan) (R; “agricolae”). To test pairwise relationships among stomatal traits, induction speed, and carbon gain during photosynthetic induction, we conducted Pearson's correlation analysis for each habitat.

To determine the most important factors affecting *C*_indu_, *C*_relax_ and *C*_total_, we examined a full model consisting of stomatal traits, response speed, average *gs*, and average intrinsic water-use efficiency during light induction and relaxation to detect the most important factors. Best regressions were selected using stepwise regression (R; “MASS”). All analyses were performed using R v. 4.2.1 (R Core Team, 2020).

## 3 Results

### 3.1 Light response curve

There was a significant difference in the parameters of the light response curve among the three species in the different habitats (Table S1). *Tithonia* *diversifolia* had higher LSP (1682.00 ± 38.83) and LCP (40.67 ± 2.17) than the two native species in sunny habitat (Fig. S1). Habitat also affected the LSP and LCP of the three species (Table S1)*.* In shady habitat, LSP (1276.37 ± 49.24) and LCP (19.33 ± 6.48) of *T. diversifolia* and LSP (1096.01 ± 108.96) of *C. bungei* were obviously lower relative to the sunny habitats. For LCP (full-sun:26.00 ± 1.16; shady: 25.33 ± 1.69) of *C. bungei*, there was no significant difference between the sunny and shady habitats. Therefore, in the shady habitats, *T. divesifolia* showed a similar LCP to but higher LSP than the two native species (Fig. S1).

### 3.2 Gas exchange parameters

**Fig. 1:**
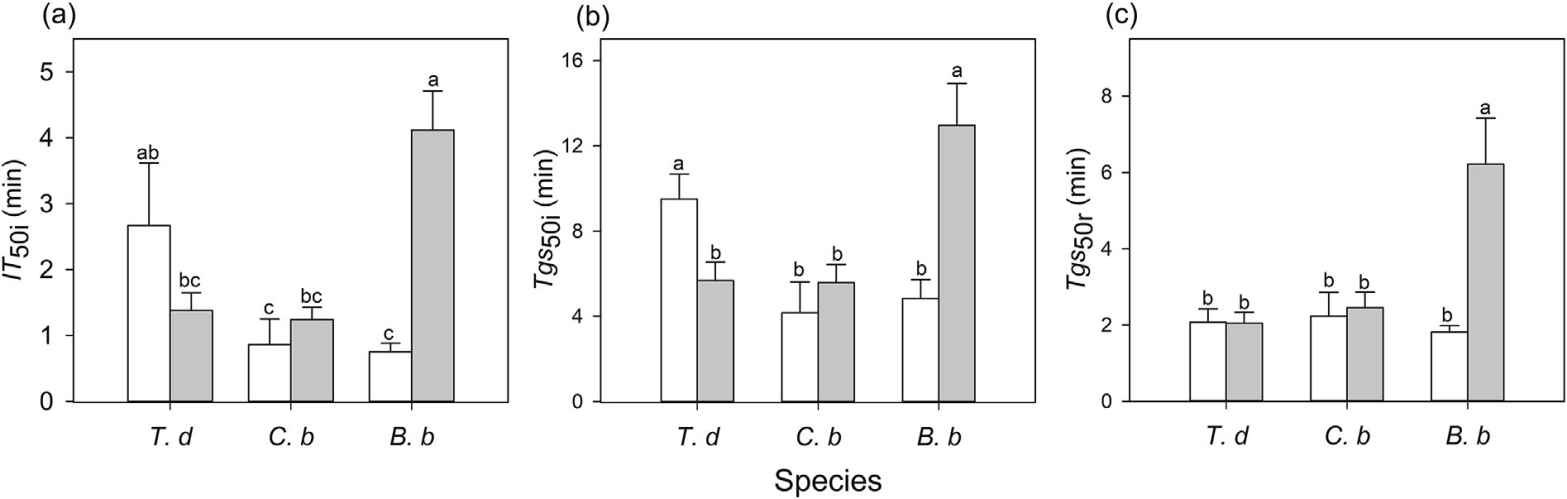
Time to 50% induction of CO_2_ uptake (*IT*_50i_, **a**), time to 50% induction of stomatal conductance (*Tgs*_50i_, **b**) during photosynthetic induction, and time to 50% relaxation of stomatal conductance (*Tgs*_50r_, **c**). These traits were measured in three species [*Tithonia diversifolia* (*T. d*), *Clerodendrum bungei* (*C. b*), and *Blumea balsamifera* (*B. b*)] in full-sun (white bar) and shady (gray bar) habitats. Each bar is the mean (±SE) of six plants. Different lowercase letters indicate statistically significant differences at *P* < 0.05.

**Fig. 2:**
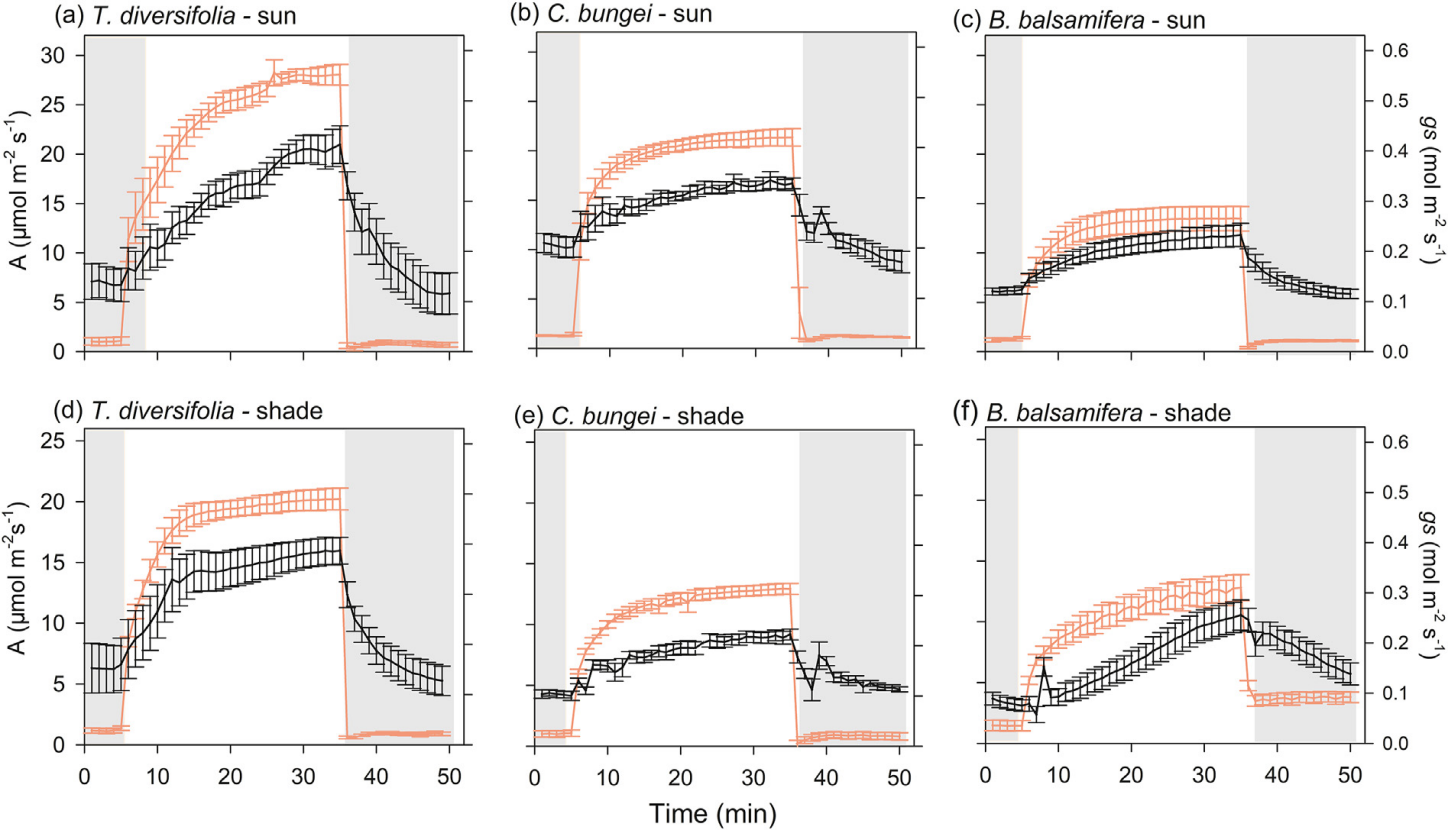
Photosynthetic rate (*A*) and stomatal conductance (*gs*) over time showing an increase during photosynthetic induction (transition from low light (50 μmol m^−2^ s^−1^) to high light (1700 μmol m^−2^ s^−1^) and photosynthetic relaxation (transition from high light to low light). Measurements were taken in three species [*Tithonia diversifolia* (**a, d**), *Clerodendrum bungei* (**b, e**) and *Blumea balsamifera* (**c, f**)] in full-sun and shady habitats. Red line and black line represent *A* and *gs*, respectively. Periods of low light are shown by the gray areas in the figure, and the period of highlight is shown in white.

**Fig. 3:**
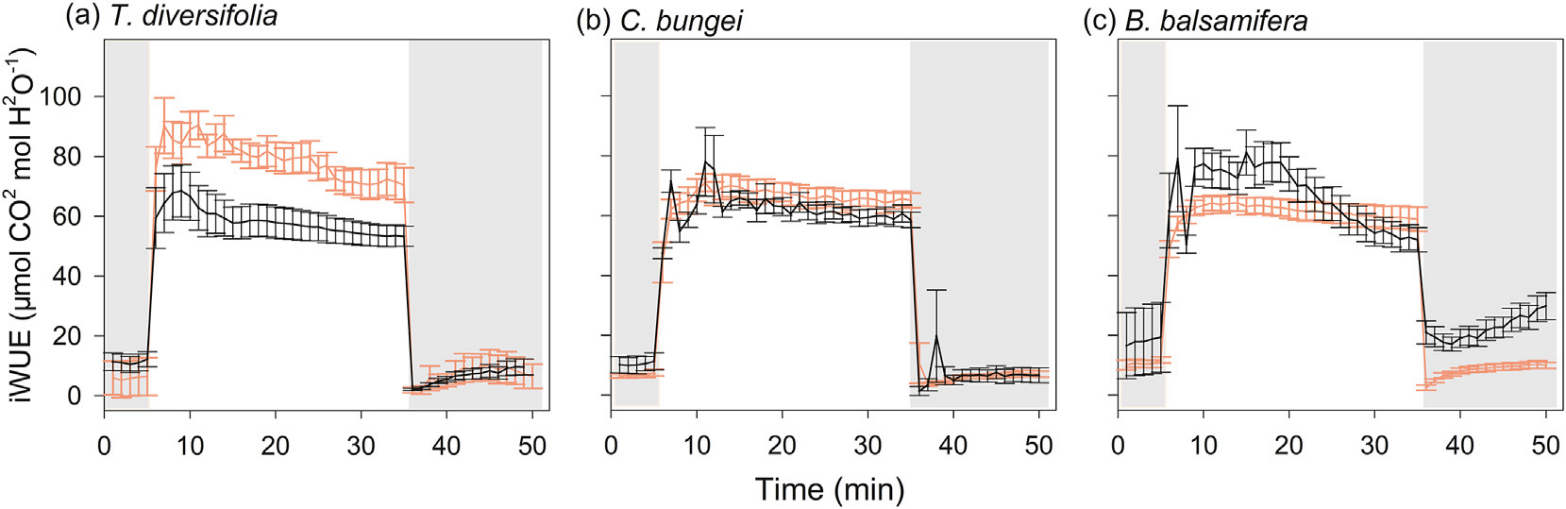
Intrinsic water-use efficiency (iWUE) over time during photosynthetic induction (transition from low light (50 μmol m^−2^ s^−1^) to high light (1700 μmol m^−2^ s^−1^) and photosynthetic relaxation (transition from high light to low light). Measurements were taken in three species [*Tithonia diversifolia* (**a**), *Clerodendrum bungei* (**b**), and *Blumea balsamifera* (**c**)] in the full-sun (red line) and shady (black line) habitats. Periods of low light are shown by the gray areas in the figure, and the period of high light is shown in white.

The speed of induction differed significantly among the three species (Table S1). In sunny habitats, the speed of induction of *A* and *gs* of *T. diversifolia* were slower (higher *IT*_*5*0i_ and *Tgs*_50i_) than those in *C. bungei* and *B. balsamifera* (Fig. 1). Habitat had a significant effect on induction speed, but this effect differed among species (significant interaction) (Table S1). The time of induction and stomatal opening of *T. diversifolia* was shortened (lower *IT*_50i_ and *Tgs*_50i_), but that of *B. balsamifera* was lengthened (higher *IT*_50i_ and *Tgs*_50i_) in shady habitat than in sunny habitat (Fig. 1). The trends in *gs* and *A* were tightly coupled, regardless of the species and habitat type during induction (Fig. 2). The iWUE first increased and then decreased with increasing light intensity for *T. diversifolia* in the two habitats and for *B. balsamfera* in shady habitat (Fig. 3). In *C. bungei,* iWUE rapidly increased and was maintained during photosynthetic induction (Fig. 3). *T. diversifolia* was the top-performing species for *gs*_mi_ and iWUE_mi_, and it also achieved the highest *C*_indu_, while *B. balsamifera* was the lowest-performing species in all habitats (Table 3).

**Table 3:** Stomatal conductance, intrinsic water-use efficiency, and carbon gain.

Habitat	Species	Photosynthetic induction			Photosynthetic relaxation			*C*_total_
		*gs*_mi_	iWUE_mi_	*C*_indu_	*gs*_mr_	iWUE_mr_	*C*_relax_	
Full sun	*T. diversifolia*	0.31 ± 0.03^a^	79.04 ± 4.24^a^	(42.65 ± 1.70) 10^3a^	0.18 ± 0.04^a^	7.07 ± 3.65^b^	(0.63 ± 0.21) 10^3b^	(43.28 ± 1.67) 10^3a^
	*C. bungei*	0.30 ± 0.01^a^	64.43 ± 2.31^bc^	(34.76 ± 0.98) 10^3b^	0.21 ± 0.01^a^	5.49 ± 0.85^b^	(1.01 ± 0.07) 10^3b^	(35.77 ± 1.03) 10^3b^
	*B. balsamifera*	0.21 ± 0.02^b^	61.04 ± 2.83^c^	(22.48 ± 2.05) 10^3c^	0.14 ± 0.01^a^	8.27 ± 0.84^b^	(0.94 ± 0.07) 10^3b^	(23.42 ± 2.02) 10^3c^
Shade	*T. diversifolia*	0.34 ± 0.04^a^	58.23 ± 5.33^c^	(32.85 ± 1.41) 10^3b^	0.18 ± 0.02^a^	6.55 ± 1.46^b^	(0.74 ± 0.10) 10^3b^	(33.60 ± 1.43) 10^3b^
	*C. bungei*	0.19 ± 0.01^b^	62.25 ± 2.84^bc^	(20.79 ± 0.67) 10^3c^	0.14 ± 0.01^a^	6.89 ± 2.65^b^	(0.70 ± 0.25) 10^3b^	(21.49 ± 0.76) 10^3c^
	*B. balsamifera*	0.17 ± 0.02^b^	75.67 ± 7.09^ab^	(18.88 ± 1.58) 10^3c^	0.18 ± 0.02^a^	22.64 ± 3.42^a^	(3.35 ± 0.38) 10^3a^	(22.23 ± 1.55) 10^3c^

Note: Values of mean ± SE (standard error) are shown.

Different lowercase letters indicate statistically significant difference (*P* < 0.05).

See Table 2 for trait abbreviations.

During the transition from high to low light, there were no significant differences in the speed of stomatal closing (*Tgs*_50r_) among species in full-sun habitat. However, in shady habitat, *B. balsamifera* showed a slower speed of stomatal closure (higher *Tgs*_50r_) relative to that in full-sun habitat, and stomatal closure was slower than that in *T. diversifolia* and *C. bungei* in shady habitat (Fig. 1). Inconsistent with light induction, the dynamics of *A* and *gs* were not coupled, with *A* decreasing much faster during light relaxation (Fig. 2). Except for *B. balsamifera,* the iWUE also rapidly decreased during photosynthetic relaxation (Fig. 3). For *B. balsamifera*, the iWUE showed an increasing trend during photosynthetic relaxation (Fig. 3). The *gs*_mr_ was not significantly different among species, but the iWUE_mr_ and *C*_relax_ of *B. balsamifera* were higher than those of the other two species during photosynthetic relaxation (Table 3). Even so, *T. diversifolia* had the highest *C*_total_ during the entire light fluctuation period (Table 3).

### 3.3 Leaf traits

**Fig. 4:**
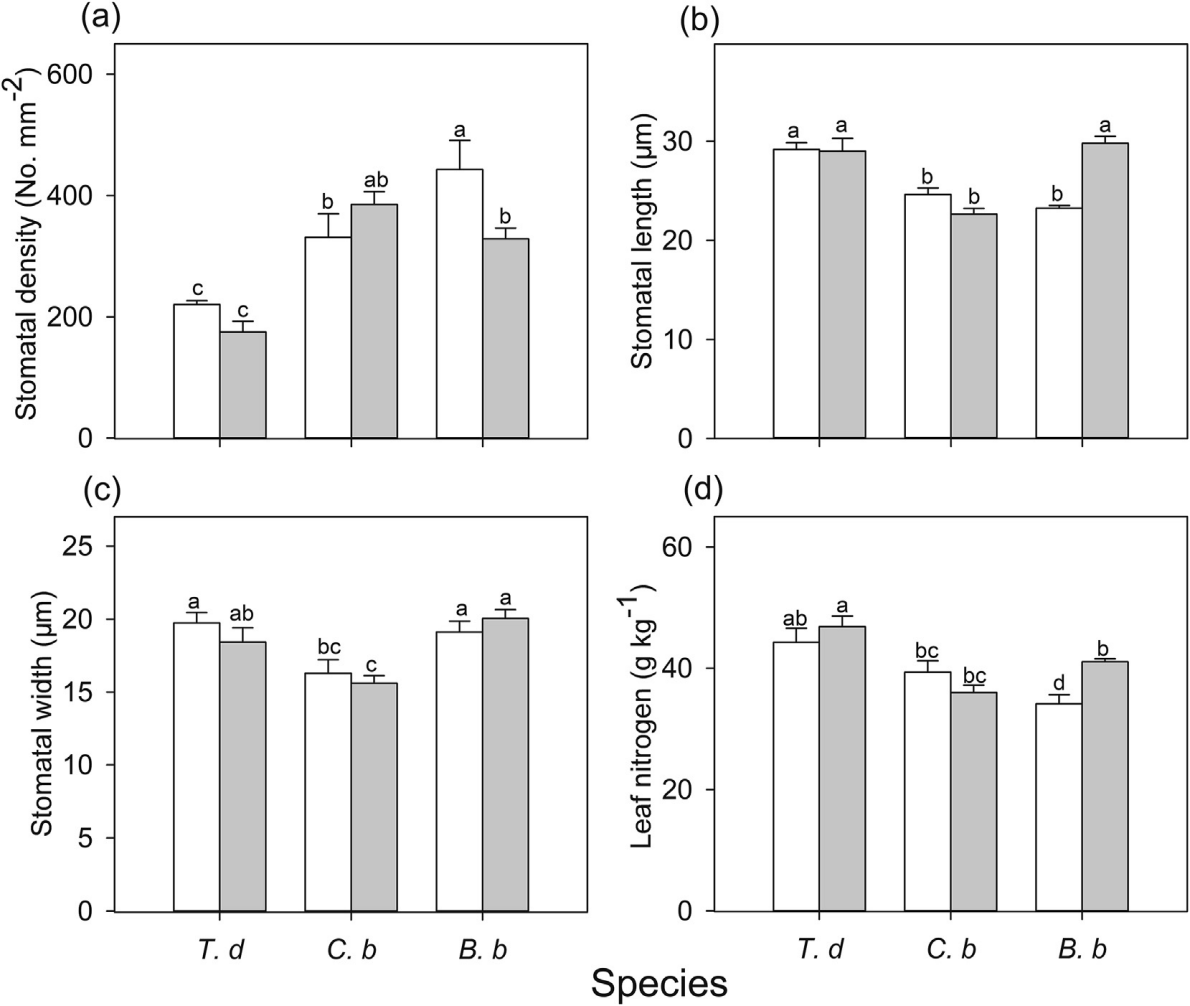
Stomatal density (**a**), stomatal length (**b**) and stomatal width (**c**), and leaf nitrogen (**d**) of three species [*Tithonia diversifolia* (*T. d*), *Clerodendrum bungei* (*C. b*) and *Blumea balsamifera* (*B. b*)] in full-sun (white bar) and shady (gray bar) habitats. Each bar is the mean (±SE) of six plants. Different lowercase letters indicate statistically significant differences at *P* < 0.05.

**Fig. 5:**
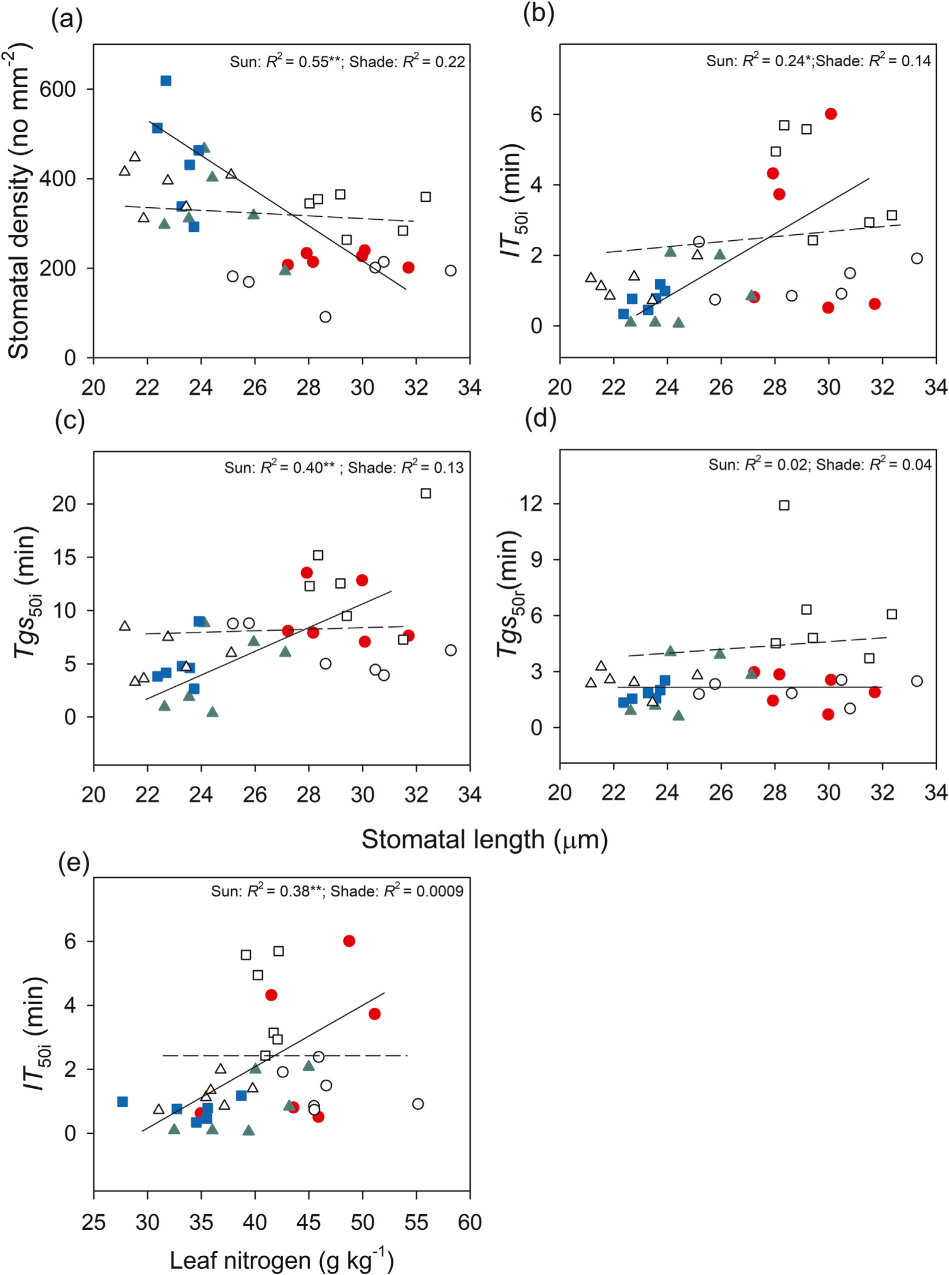
Correlations between stomatal length and stomatal density (**a**), time to 50% induction of CO_2_ uptake (*IT*_50i_, **b**), time to 50% induction of stomatal conductance (*Tgs*_50i_, **c**) during photosynthetic induction, and time to 50% relaxation of stomatal conductance (*Tgs*_50r_, **d**) and correlation between leaf nitrogen and time to 50% induction of CO_2_ uptake (**e**) in three species [*Tithonia diversifolia* (full-sun: red solid circle; shade: empty circle), *Clerodendrum bungei* (full-sun: green solid triangle; shade: empty triangle), and *Blumea balsamifera* (full-sun: blue solid square; shade: empty square)] in the full-sun (full line) and shady (dotted line) habitats. Each point represents one replication.

Leaf traits differed among the species and habitats (Table S1). In full-sun habitats, *T. diversifolia* and *C. bungei* had higher leaf nitrogen than *B. balsamifera*. In shady habitats, *B. balsamifera* had increased leaf nitrogen relative to that in sunny habitats, but both *C. bungei* and *B. balsamifera* had lower leaf nitrogen than *T. diversifolia* (Fig. 4). Stomatal traits were different among species and habitats (Table S1). Regardless of the habitat type, *T. diversifolia* had the lowest stomatal density (Fig. 4). Compared to the sunny habitats, the stomatal density of *B. balsamifera* was significantly lower in shady habitats (Fig. 4). In full-sun habitats, *T. diversifolia* had higher stomatal length values than the two native species, and a higher stomatal width than *C. bungei* (Fig. 4). *B. balsamifera* displayed longer stomatal length in shady habitat than in full-sun habitat. In shady habitat, the stomatal size of *B. balsamifera* was higher than that of *C. bungei*, but similar to that of *T. diversifolia* (Fig. 4). There was also a significant negative relationship between stomatal length and density in full-sun habitats (Fig. 5).

### 3.4 Correlations between gas change parameters and leaf traits

**Fig. 6:**
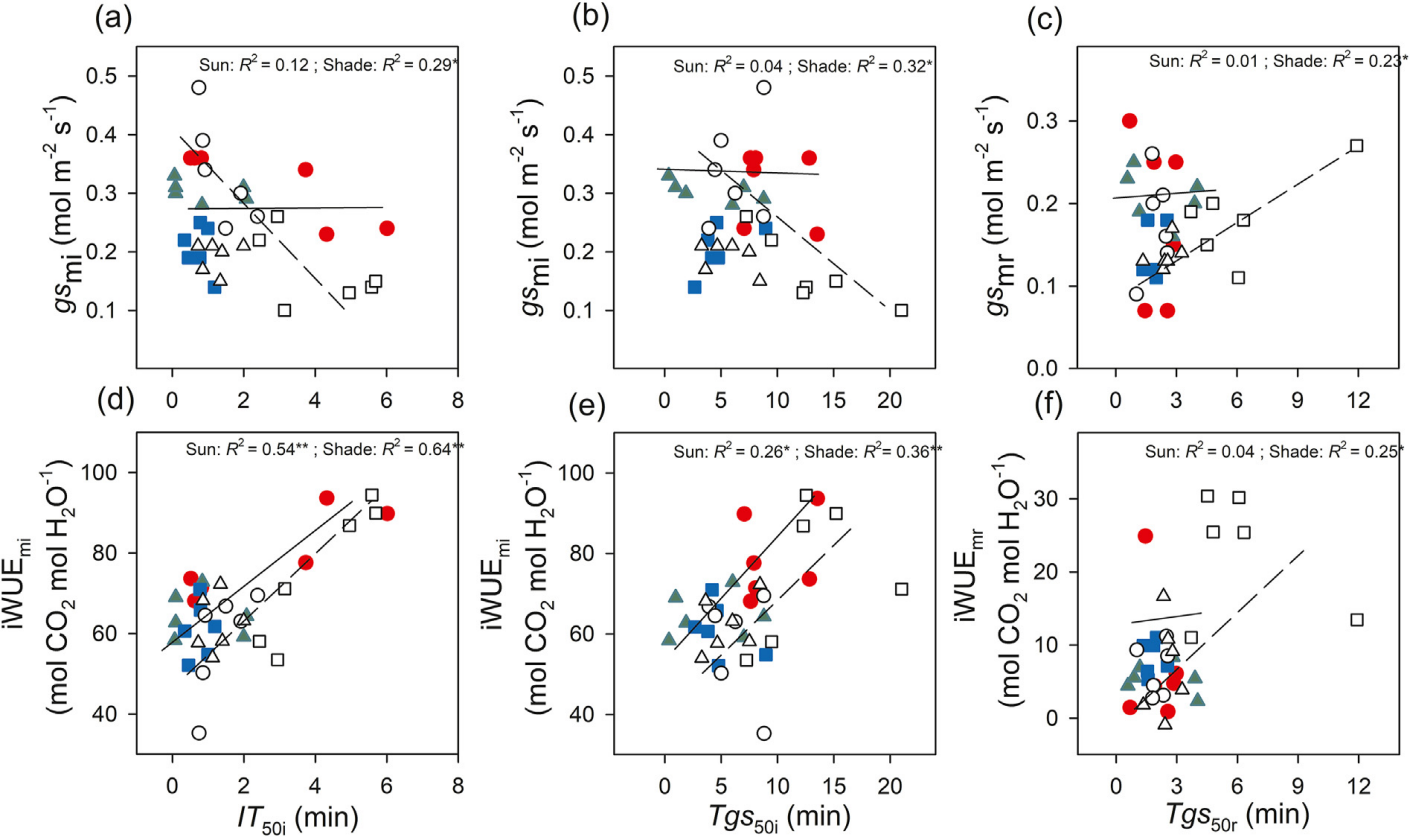
Correlations between average stomatal conductance and time to 50% induction of CO_2_ uptake (*IT*_50i_, **a**), time to 50% induction of stomatal conductance (*Tgs*_50i_, **b**) during induction, and time to 50% relaxation of stomatal conductance (*Tgs*_50r_, **c**) during relaxation. Correlations between average intrinsic water-use efficiency and *IT*_50i_ (**d**), *Tgs*_50i_ (**e**), and *Tgs*_50r_ (**f**) in three species [*Tithonia diversifolia* (full-sun: red solid circle; shade: empty circle), *Clerodendrum bungei* (full-sun: green solid triangle; shade: empty triangle) and *Blumea balsamifera* (full-sun: blue solid square; shade: empty square)] in full-sun (full line) and shady (dotted line) habitats. Each point represents one replication.

Induction speed (*IT*_50i_ increased) decreased with increasing leaf nitrogen, and these relationships were observed in sunny habitats. With increasing stomatal length, the induction speed (*IT*_50i_ and *Tgs*_50i_ increased) decreased in full-sun habitats; however, no such relationships were found in shady habitats (Fig. 5). Stomatal length had no obvious relationship with *Tgs*_50r_ in either habitat (Fig. 5). No significant correlations were found between stomatal density and induction speed (in full-sun habitats, *IT*_50i_: *R*^2^ = 0.09, *P* = 0.220; *Tgs*_50i_: *R*^2^ = 0.11, *P* = 0.181; *Tgs*_50r_: *R*^2^ = 0.008, *P* = 0.720; in shady habitats: *IT*_50i_: *R*^2^ = 0.08, *P* = 0.244; *Tgs*_50i_: *R*^2^ = 0.07, *P* = 0.318; *Tgs*_50r_: *R*^2^ = 0.13, *P* = 0.183), although there was a negative relationship between stomatal length and density. The induction time (*IT*_50i_ and *Tgs*_50i_) showed significantly negative relationships with *gs*_mi_ in shady habitat but not in full-sun habitat (Fig. 6). However, induction time (*IT*_50i_ and *Tgs*_50i_) showed significant positive relationships with iWUE_mi_ in both habitats (Fig. 6). The relaxation time (*Tgs*_50r_) showed a significantly positive relationship with *gs*_mr_ and iWUE_mr_ in shady habitat but not in full-sun habitat (Fig. 6).

### 3.5 Modelling trait variation

In full-sun habitat, *C*_indu_ was best predicted by *gs*_mi_ and iWUE_mi_; *C*_relax_ was best predicted by leaf traits (leaf nitrogen, stomatal density and length) and iWUE_mr_; *C*_total_ was best predicted by *gs*_mi_ and iWUE_mi_ (Table 4). In shady habitat, *C*_indu_ was best predicted by *IT*_50i_, *gs*_mi_ and iWUE_mi_, *C*_relax_ was best predicted by *gs*_mr_ and iWUE_mr_, *C*_total_ was best predicted by *gs*_mi_, and iWUE_mi_, followed by *IT*_50i_ and *Tg*_50r_ (Table 4).

**Table 4:** Step regression models to predict carbon gain during light fluctuation.

	Full-sun habitat			Shady habitat		
	*C*_indu_	*C*_relax_	*C*_total_	*C*_indu_	*C*_relax_	*C*_total_
***IT***_**50i**_	−0.08^#^	–	−0.08^∗∗^	−0.31^∗^	–	−0.46^∗^
***Tgs***_**50i**_	–	–	–	–	–	0.17^#^
***Tgs***_**50r**_	–	0.33^∗^	–	–	–	−0.41^∗^
***gs***_**mi**_	0.79^∗∗∗^	–	0.75^∗∗∗^	1.11^∗∗∗^	–	0.87^∗∗^
***gs***_**mr**_	–	0.31^#^	–	–	0.37^∗∗∗^	0.32^#^
**iWUE**_**mi**_	0.5^∗∗∗^	–	0.53^∗∗∗^	0.56^∗∗^	–	0.73^∗∗^
**iWUE**_**mr**_	–	0.52^∗∗^	−0.06^∗∗^	–	0.89^∗∗∗^	0.20^∗^
**SL**	–	−0.70^∗^	–	–	–	0.39^∗^
**SD**	–	−0.58^∗^	−0.07^∗∗^	–	0.11^#^	0.27^∗^
**LN**	0.06^#^	−0.50^∗^	–	–	–	0.15^#^
**Model *R***^***2***^	0.99	0.78	0.99	0.94	0.97	0.99

Note: Standardized beta value from the final equation is reported only for significant predictors.

See Table 2 for trait abbreviations.

^#^ P < 0.10; ^∗^*P* < 0.05; ^∗∗^*P* < 0.01; ^∗∗∗^*P* < 0.001.

## 4 Discussion

To understand how the invasive plant *Tithonia* *diversifolia* responds to light fluctuation, we compared the photosynthetic responses to fluctuating light among the invasive plant and two native plants distributed in full-sun and shady environments. We found that the photosynthetic induction dynamics differed among the three species in different habitats. The invasive species *T. diversifolia* showed different photosynthetic induction in full-sun and shady habitats, whereas the native species *B. balsamifera* displayed different photosynthetic relaxations. For *C. bungei*, the photosynthetic dynamics were not significantly different between the two habitats. These differences lead to an overall higher carbon gain during fluctuating light for the invasive species *T. diversifolia* in both habitats, which may contribute to its successful invasion.

### 4.1 Difference in photosynthetic dynamic

*Tithoniadiversifolia* adopts different strategies to respond to light induction in full-sun and shady habitats to maximize carbon gain during light fluctuation. In full-sun habitat, *T. diversifolia* showed slower photosynthetic induction speed in *gs* and *A* than native species (Fig. 1). This is attributed to the larger stomata of *T. diversifolia* relative to native species, as suggested by positive relationship between stomatal length and induction speed in full-sun habitat (Fig. 5). Stoma controls both CO_2_ uptake and water transpiration (Voelker et al., 2016); when the light intensity increases suddenly, the rapid opening of large stomata may obtain more CO_2_, but potentially lead to more water loss, leading to a reduction in the photosynthesis rate (Drake et al., 2013). In the field, wilting and drooping leaves have been observed in *T. diversifolia* at noon, suggesting that this invasive species may be less resistant to drought than the two native species. Correspondingly, we also observed that *T. diversifolia* had higher iWUE_mi_ than the two native species in full-sun habitat, which contributed to higher *C*_indu_ (Tables 3 and 4). This is inconsistent with the idea that a slow response to photosynthetic induction may result in a loss of daily carbon gain (Taylor and Long, 2017; Tanaka et al., 2019). On the contrary, in shady habitat, *T. diversifolia* increased the induction speed relative to conspecifics in full-sun habitat, and it was faster than that of the native plant *B. balsamifera*. Thus, faster induction speed plays a key role in maintaining high *gs*_mi_ in shady habitat (Fig. 6) and contributes significantly to carbon gain during photosynthetic induction in C_indu_ (Table 4). Although the increase in induction speed led to some loss of iWUE_mi_, this appears to be of less importance in such an environment. The regression analysis results also indicated that iWUE_mi_ is a key factor contributing to carbon gain during light induction in full-sun habitat, whereas *gs*_mi_ plays an important role in carbon gain during light induction in shady habitat (Table 4).

Light fleck theory predicts that shade-adapted plants should rapidly open stomata in response to light increase to make use of light flecks, while also slowly closing stomata during light decrease to make the most of potential future light flecks (Knapp and Smith, 1987). This theory explains the difference in stomata closure speed among the three species in shady habitat. The native species *B. balsamifera* had lower LSP and LCP values than the other two species, especially in shady habitat (Fig. S1), suggesting that it is more adapted to shady environment than the other two species. Correspondingly, we found that this species showed slower stomata closure (higher *Tgs*_50r_ value) from high light to low light (Fig. 1). In addition, the slower closing of stomata may maintain *gs* to some degree and promote photosynthesis. We also observed that *B. balsamifera* displayed a higher *gs*_mr_ and iWUE_mr_ (Table 3), which contributed to a higher *C*_relax_ (Table 4). One possible reason for this is that water is not a limiting factor in shady habitat. Maximum utilization of light energy may be more important in such environments. Nevertheless, the strategy adopted by *T. diversifolia* in different habitats was more effective for total carbon gain relative to that of *B. balsamifera*, because total carbon gain during light fluctuation mainly came from photosynthetic induction rather than photosynthetic relaxation.

### 4.2 Effect of leaf traits

Stomatal traits are important factors that affect the photosynthetic response dynamics to light fluctuations (Xiong et al., 2022). Our results also indicated relationships between stomatal length and stomatal opening and induction speed; species with large stomata have slower stomatal opening relative to species with small stomata. This is consistent with other studies on many species (Drake et al., 2013; Xiong et al., 2022). However, a relationship between stomatal length and stomatal opening speed was not observed in shady habitat. This could be because the stomatal length of *B. balsamifera* was significantly higher in shady habitat than in full-sun habitat, whereas there was no significant difference in stomatal length between the two habitats for the other two species. Thus, changes in stomatal length may lead to slower stomatal opening and closing speeds in *B. balsamifera* during light induction and relaxation (Figs. 1 and 4), suggesting that stomatal morphological traits mediate stomatal behavior species. Drake et al. (2013) also suggested that plants with larger stomata often exhibit a slower response rate to environmental fluctuations (Drake et al., 2013). However, the specific regulatory mechanism for stomatal morphology is still not clear. One possible mechanism is that larger stomata may require more osmotic substances and energy inside the leaves than smaller stomata (Santelia and Lawson, 2016). In addition, *T. diversifolia* showed faster induction and stomatal opening speed in shady habitat than in full-sun habitat, although no significant differences in stomatal traits were found. This suggests that, in addition to stomatal traits, there may be other factors or mechanisms affecting the induction speed of *T. diversifolia*.

Leaf nutrients are essential elements for stromal enzymes and thylakoid proteins (Sudo et al., 2003; Takashima et al., 2004), and can potentially affect photosynthesis. Liu et al. (2021) found that leaf nitrogen content was negatively correlated with the time to 50% and 100% of the maximum photosynthetic rate across eight genotypes of *Brassica napus*. Sun et al. (2022) also indicated that tomato seedlings grown under high nitrogen conditions had faster photosynthetic induction speeds. In contrast, in this study, we found a positive relationship between leaf nitrogen content and time to 50% of the maximum photosynthetic rate in full-sun habitats. One possible reason for this is that plants with high leaf nitrogen also have a larger stomatal size (longer stomatal length). Stomatal traits have a greater impact than leaf nitrogen content on photosynthetic induction speed.

## Author contributions

J.L. designed and performed the experiments, analyzed the results, and wrote the paper. S.-B.Z. designed and performed the experiments. Y.-P. L. contributed to design, analysis and writing of the paper.

## Declaration of competing interest

We have no known competing financial interests or personal relationships that could have influenced the work reported in this study.

## Data availability statement

The data used in this study will be uploaded to the Science DB database to be shared when the manuscript is accepted.

## References

[bib1] Dai G.H., Wang S., Geng Y.P. (2021). Potential risks of *Tithonia diversifolia* in Yunnan Province under climate change. Ecol. Res..

[bib2] Deans R.M., Brodribb T.J., Busch F.A. (2019). Plant water–use strategy mediates stomatal effects on the light induction of photosynthesis. New Phytol..

[bib3] Diagne C., Leroy B., Vaissière A.C. (2021). High and rising economic costs of biological invasions worldwide. Nature.

[bib4] Drake P.L., Froend R.H., Franks P.J. (2013). Smaller, faster stomata: scaling of stomatal size, rate of response, and stomatal conductance. J. Exp. Bot..

[bib5] Elliott-Kingston C., Haworth M., Yearsley J.M. (2016). Does size matter? Atmospheric CO_2_ may be a stronger driver of stomatal closing rate than stomatal size in taxa that diversified under low CO_2_. Front. Plant Sci..

[bib6] Kaiser E., Kromdijk J., Harbinson J. (2017). Photosynthetic induction and its diffusion, carboxylation and electron transport processes as affected by CO_2_ partial pressure, temperature, air humidity and blue irradiance. Ann. Bot..

[bib7] Kaiser E., Morales A., Harbinson J. (2016). Metabolic and diffusional limitations of photosynthesis in fluctuating irradiance in *Arabidopsis thaliana*. Sci. Rep..

[bib8] Kato-Noguchi H. (2020). Involvement of allelopathy in the invasive potential of *Tithonia diversifolia*. Plants.

[bib9] Knapp A.K., Smith W.K. (1987). Stomatal and photosynthetic responses during sun/shade transitions in subalpine plants: influence on water use efficiency. Oecologia.

[bib10] Laduke J. (1982). Revision of *Tithonia*. Rhodora.

[bib11] Lawson T., Blatt M.R. (2014). Stomatal size, speed, and responsiveness impact on photosynthesis and water use efficiency. Plant Physiol..

[bib12] Liu W.J., Zhang Y.P., Li H.M. (2004). Fog characteristics in a tropical seasonal rain forest in Xishuangbanna. Chin. J. Plant Ecol..

[bib13] Liu J., Zhang J., Estavillo G.M. (2021). Leaf N content regulates the speed of photosynthetic induction under fluctuating light among canola genotypes (*Brassica napus* L.). Physiol. Plantarum.

[bib14] Morales E. (2000). Estimating phylogenetic inertia in *Tithonia* (Asteraceae): a comparative approach. Evolution.

[bib15] Mott K.A., Woodrow I.E. (2000). Modelling the role of Rubisco activase in limiting non-steady-state photosynthesis. J. Exp. Bot..

[bib16] Obiakara M.C., Fourcade Y. (2018). Climatic niche and potential distribution of *Tithonia diversifolia* (Hemsl.) A. Gray in Africa. PLoS One.

[bib17] Otusanya O.O., Ilori O. (2012). Phytochemical screening and the phytotoxic effects of aqueous extracts of *Tithonia diversifolia* (Hemsl.) A. Gray. Int. J. Biol..

[bib18] Oyerinde R.O., Otusanya O.O., Akpor O.B. (2009). Allelopathic effect of *Tithonia diversifolia* on the germination, growth and chlorophyll contents of maize (*Zea mays* L.). Sci. Res. Essays.

[bib19] Pearcy R.W. (1990). Sunflecks and photosynthesis in plant canopies. Annu. Rev. Plant Physiol. Plant Mol. Biol..

[bib20] Pearcy R.W. (1994).

[bib21] R Core Team (2020). https://www.R-project.org/.

[bib22] Sakoda K., Adachi S., Yamori W. (2022). Towards improved dynamic photosynthesis in C_3_ crops by utilising natural genetic variation. J. Exp. Bot..

[bib23] Santelia D., Lawson T. (2016). Rethinking guard cell metabolism. Plant Physiol..

[bib24] Slattery R.A., Walker B.J., Weber A.P.M. (2018). The impacts of fluctuating light on crop performance. Plant Physiol..

[bib25] Soleh M.A., Tanaka Y., Kim S.Y. (2017). Identification of large variation in the photosynthetic induction response among 37 soybean [*Glycine max* (L.) Merr.] genotypes that is not correlated with steady-state photosynthetic capacity. Photosynth. Res..

[bib26] Sudo E., Makino A., Mae T. (2003). Differences between rice and wheat in ribulose-1,5-bisphosphate regeneration capacity per unit of leaf-N content. Plant Cell Environ..

[bib27] Sun H., Zhang Y.Q., Zhang S.B. (2022). Photosynthetic induction under fluctuating light is affected by leaf nitrogen content in tomatoes. Front. Plant Sci..

[bib28] Takashima T., Hikosaka K., Hirose T. (2004). Photosynthesis or persistence: nitrogen allocation in leaves of evergreen and deciduous Quercus species. Plant Cell Environ..

[bib29] Tanaka Y., Adachi S., Yamori W. (2019). Natural genetic variation of the photosynthetic induction response to fluctuating light environment. Curr. Opin. Plant Biol..

[bib30] Taylor S.H., Long S.P. (2017). Slow induction of photosynthesis on shade to sun transitions in wheat may cost at least 21% of productivity. Philos. Trans. R. Soc., B..

[bib31] Voelker S.L., Brooks J.R., Meinzer F.C. (2016). A dynamic leaf gas-exchange strategy is conserved in woody plants under changing ambient CO_2_: evidence from carbon isotope discrimination in paleo and CO_2_ enrichment studies. Global Change Biol..

[bib32] Xiong Z., Xiong D.L., Cai D.T. (2022). Effect of stomatal morphology on leaf photosynthetic induction under fluctuating light across diploid and tetraploid rice. Environ. Exp. Bot..

[bib33] Zhang Q., Peng S.B., Li Y. (2019). Increased rate of light-induced stomatal conductance is related to stomatal size in the *Oryza* genus. J. Exp. Bot..

[bib34] Zhu X.G., Ort D., Whitmarsh J. (2004). The slow reversibility of photosystem II thermal energy dissipation on transfer from high to low light may cause large losses in carbon gain by crop canopies: a theoretical analysis. J. Exp. Bot..

