# Supplementary material for: Photosynthetic response dynamics in the invasive species Tithonia diversifolia and two co-occurring native shrub species under fluctuating light conditions
Source: Plant Divers. 2023 Apr 25;46(2):265–73. doi: 10.1016/j.pld.2023.04.001 (PMC11128914; doi:10.1016/j.pld.2023.04.001)
Supplement: Multimedia component 1 [file mmc1.docx]

**Photosynthetic response dynamics in the invasive species *Tithonia diversifolia* and two co-occurring native shrub species under fluctuating light conditions**

Ju Li ^a, b^, Shu-Bin Zhang ^a^, Yang-Ping Li ^a,^*

**^a^ *CAS Key Laboratory of Tropical Forest Ecology, Xishuangbanna Tropical Botanical Garden, Chinese Academy of Sciences, Menglun, Mengla 666303, Yunnan, China***

**^b^ *University of Chinese Academy of Sciences, Beijing 100049, China***

* Corresponding author.

1. *mail address: liyp@xtbg.org.cn (Y.-P. Li)*

**Table S1 Results of the** two-way analysis of variance (ANOVA) for all variables.

|  | **Traits** | ***df*** | **Habitat (H)** | | ***df*** | **Species (S)** | | ***df*** | **H × S** | |
| --- | --- | --- | --- | --- | --- | --- | --- | --- | --- | --- |
|  |  |  | F**-value** | P**-value** |  | F**-value** | P**-value** |  | F**-value** | P**-value** |
| **Photosynthetic traits** | **LCP** | (1,30) | 9.52 | **<0.001** | (2,30) | 18.01 | **<0.001** | (2,30) | 5.05 | **0.013** |
|  | **LSP** | (1,30) | 30.25 | **<0.001** | (2,30) | 20.33 | **<0.001** | (2,30) | 1.15 | 0.33 |
|  | **IT**50i | (1,30) | 3.95 | 0.056 | (2,30) | 3.99 | **0.029** | (2,30) | 10.86 | **<0.001** |
|  | ***Tgs***50i | (1,29) | 3.93 | 0.057 | (2,29) | 5.32 | **0.011** | (2,29) | 10.67 | **<0.001** |
|  | ***Tgs***50r | (1,27) | 9.74 | **0.004** | (2,27) | 5.49 | **0.010** | (2,27) | 7.45 | **0.003** |
|  | ***gs***mi | (1,30) | 5.58 | **0.025** | (2,30) | 19.28 | **<0.001** | (2,30) | 4.31 | **0.023** |
|  | ***gs***mr | (1,30) | 0.32 | 0.574 | (2,30) | 0.33 | 0.720 | (2,30) | 3.31 | 0.050 |
|  | **iWUE**mi | (1,30) | 0.59 | 0.448 | (2,30) | 0.90 | 0.417 | (2,30) | 7.98 | **0.002** |
|  | **iWUE**mr | (1,30) | 6.50 | **0.016** | (2,30) | 8.98 | **0.001** | (2,30) | 5.49 | **0.009** |
|  | ***C***indu | (1,30) | 57.67 | **<0.001** | (2,30) | 68.00 | **<0.001** | (2,30) | 6.30 | **0.005** |
|  | ***C***relax | (1,30) | 18.19 | **<0.001** | (2,30) | 28.54 | **<0.001** | (2,30) | 23.89 | **<0.001** |
|  | ***C***total | (1,30) | 48.95 | **<0.001** | (2,30) | 57.82 | **<0.001** | (2,30) | 10.24 | **<0.001** |
| **Stomatal traits** | **SD** | (1,30) | 2.25 | 0.144 | (2,30) | 25.15 | **<0.001** | (2,30) | 4.39 | **0.021** |
|  | **SL** | (1,30) | 5.54 | **0.025** | (2,30) | 25.71 | **<0.001** | (2,30) | 17.35 | **<0.001** |
|  | **SW** | (1,30) | 0.35 | 0.560 | (2,30) | 13.26 | **<0.001** | (2,30) | 1.14 | 0.333 |
|  | **LN** | (1.27) | 0.93 | 0.34 | (2,27) | 15.64 | **<0.001** | (2,27) | 3.73 | **0.04** |

Note: Bold value indicates statistically significant difference (*P* < 0.05).


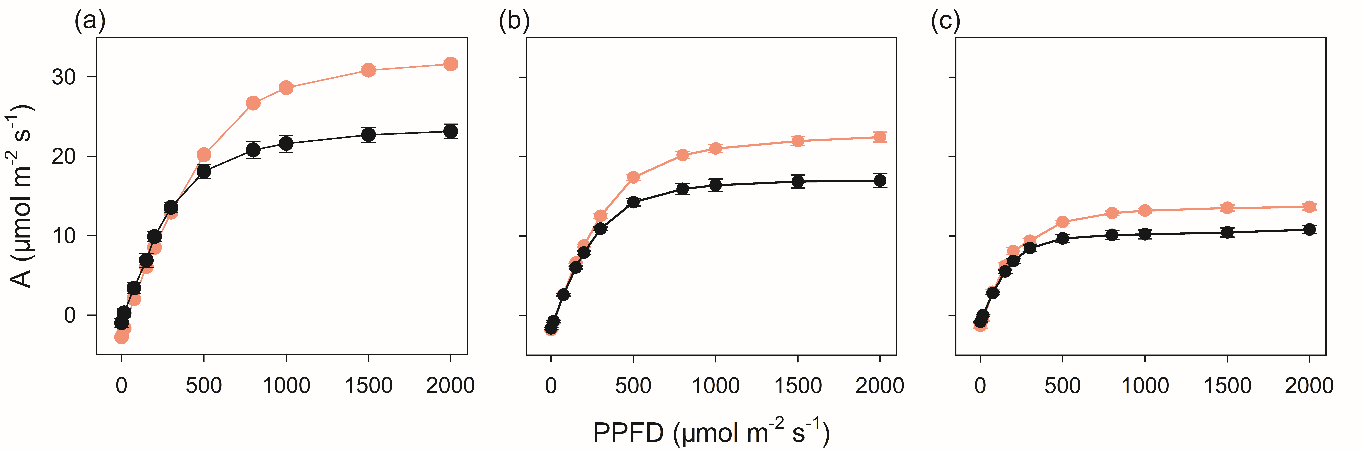


**Fig. S1.** Response of photosynthetic rate (*A*) to light intensity in three species [*Tithonia diversifolia* (**a**), *Clerodendrum bungei* (**b**), and *Blumea balsamifera* (**c**)] in the full-sun (red line) and shady (black line) habitats. Each point is the mean (±SE) of six plants (n = 6).
